# Enhanced xylitol production using immobilized *Candida tropicalis* with non-detoxified corn cob hemicellulosic hydrolysate

**DOI:** 10.1007/s13205-016-0388-8

**Published:** 2016-02-16

**Authors:** Tatyaso Yewale, Shruti Panchwagh, Srinivasan Rajagopalan, Pradip B. Dhamole, Rishi Jain

**Affiliations:** 1 Praj Matrix R & D Center, Division of Praj Industries Ltd., 402/403/1098, Urawade, Pune, Maharashtra 412115 India; 2 Department of Technology, Savitribai Phule Pune University, Ganeshkhind Road, Pune, Maharashtra 411007 India; 3 Chemical Engineering Department, Visvesvaraya National Institute of Technology, South Ambazari Road, Nagpur, Maharashtra 440010 India

**Keywords:** *Candida tropicalis*, Immobilization, Calcium alginate, Corn cob hemicellulosic hydrolysate

## Abstract

This study reports an industrially applicable non-sterile xylitol fermentation process to produce xylitol from a low-cost feedstock like corn cob hydrolysate as pentose source without any detoxification. Different immobilization matrices/mediums (alginate, polyvinyl alcohol, agarose gel, polyacrylamide, gelatin, and *κ*-carrageenan) were studied to immobilize *Candida tropicalis* NCIM 3123 cells for xylitol production. Amongst this calcium alginate, immobilized cells produced maximum amount of xylitol with titer of 11.1 g/L and yield of 0.34 g/g. Hence, the process for immobilization using calcium alginate beads was optimized using a statistical method with sodium alginate (20, 30 and 40 g/L), calcium chloride (10, 20 and 30 g/L) and number of freezing–thawing cycles (2, 3 and 4) as the parameters. Using optimized conditions (calcium chloride 10 g/L, sodium alginate 20 g/L and 4 number of freezing–thawing cycles) for immobilization, xylitol production increased significantly to 41.0 g/L (4 times the initial production) with corn cob hydrolysate as sole carbon source and urea as minimal nutrient source. Reuse of immobilized biomass showed sustained xylitol production even after five cycles.

**Electronic supplementary material:**

The online version of this article (doi:10.1007/s13205-016-0388-8) contains supplementary material, which is available to authorized users.

## Introduction

The increasing demand and exorbitant cost of low calorie polyol like xylitol open up challenge for making low-cost xylitol from renewable feedstock. Xylitol, a naturally occurring sugar alcohol sweetener, that has sweetness similar to sucrose but 40 % lower energy, negative heat of dissolution, low viscosity in solution, absence of the Maillard reaction, higher chemical stability, and several biomedical properties (Bär et al. 1991). Emil Fisher was the first to synthesize xylitol by reacting xylose with sodium amalgam in 1891 (Bär et al. 1991). Xylitol is beneficial for nutrition (Sreenivas-Rao et al. 2006), for prevention of dental caries (Emidi 1978) and low-calorie food preparation for diabetic patients (Pepper and Olinger 1988) but these applications are limited due to high cost of xylitol produced by chemical means. The selling cost for xylitol is 6–7 $/kg for bulk purchase (Rafiqul and Sakinath 2013). Xylitol is currently manufactured by chemical hydrogenation of pure d-xylose in the presence of nickel catalyst at elevated temperature and pressure, yielding a product with a high purity (>99.5 %) and a yield of 50–60 % with respect to the initial xylose (Dieters 1975; Ojamo et al. 2009). Alternatively, xylitol can be produced by biological process which shows certain advantages like milder conditions of pressure, temperature, pH, agitation, cell inhibitors and lower costs of downstream processing due to the production of lower amounts of by-products (Saha 2003). However, carbon source and operating cost must be economically competitive to ensure the feasibility of the process. Corn cob, a major waste obtained in corn production and is a promising and attractive alternative for xylose rich hemicellulosic hydrolysate stream (Rivas et al. 2003).

While comparing with the free cell system, the immobilized microorganism system can be used to improve the fermentation performance and to reduce the overall production costs (Roberto et al. 1991). Immobilized cell bioprocesses becomes obvious and most preferred solution because they allow higher fermentation rates, permit high cell concentration, reuse of cells for extended time, reduce costs related to inoculum development, protect the entrapped biocatalyst from inhibitors, prevent washout and provide ease of separation of biocatalyst from fermentation broth (Jirku et al. 2000). Cell immobilization via gel entrapment is widely used in bench-scale tests, and many gel-like materials are used as carriers, which may be based on natural (alginate, *κ*-carrageenan, agarose, agar, chitosan, etc.) or synthetic (polyacrylamide, polyacrylate, polyurethane, etc.) polymers or precursors (Lozinsky et al. 1997). Among these supports screened for yeast cell immobilization to perform xylose-to-xylitol bioconversion, calcium alginate support has received more attention in biomedical and food industries due to properties viz. integrity, minimal mass transfer limitation, cheap, non-toxic, mild conditions and utilizes ingredients that are accepted as food additive (Champagne et al. 2000). The productivity of immobilized system is affected by three parameters, i.e. calcium chloride, sodium alginate and number of freezing–thawing cycles (Cunha et al. 2009). However, limited number of studies report optimized conditions for immobilization of *Candida* and even so, most of these studies use detoxified hydrolysate for fermentation (Wang et al. 2009; Carvalho et al. 2003, 2004; Cheng et al. 2009; El-Batal and Khalaf 2004; Liaw et al. 2008; Deng et al. 2006; Sarrouh et al. 2007; Gyan et al. 2011). Inclusion of the detoxification step results in an increase in the overall production cost. It is therefore, desirable to use non-detoxified hydrolysate. Hence, the present work was undertaken with an objective to enhance xylitol production from non-detoxified corn cob hydrolysate using statistically optimized immobilized process for entrapping the cells.

## Materials and methods

### Raw material and chemicals

Corn cob was provided by Abhyoday feedstock suppliers, Jalgaon, India. These are air-dried and milled into small particles (3.5 mm × 2.5 mm) before acid hydrolysis. Aminex column HPX87H (300 mm × 7.8 mm) was purchased from Biorad, Hercules, CA, USA. All used chemicals were of analytical grade. Sodium alginate, calcium chloride and sodium citrate were procured from Himedia, Mumbai.

### Preparation of corn cob hydrolysate

The composition of corn cob used in this study contained 3–5 % w/w of moisture, and the remaining dry matter was made up 60 % w/w of total reducing sugars, of which 25.0 % w/w hemicellulose and 35 % w/w cellulose, 23.0 % w/w lignin and 3 % w/w ash, 3.0 % w/w protein, 1.0 % w/w uronic acid and 2.0 % acetates.

Corn cob contains various components such as lignin, cellulose, hemicellulose, various extractives and inorganic components. Corn cob hydrolysate was obtained by Praj patented pretreatment procedure (Pal et al. 2013). This pretreatment involved corn cob solid of 15–20 % w/w, dilute acid concentration of 1–3 % w/w, screw speed of 2–4 rpm, reaction temperature of 160–180 °C and reaction time of 15–20 min. Then the corn cob slurry and aqueous solution after reaction were discharged, and separated in a solid–liquid separation equipment. The obtained supernatant was rich in xylose concentration, and the main composition was (w/w) xylose 4–6 %, glucose 0.5–1.0 %, arabinose 0.1–0.3 %, acetic acid 0.2–0.6 %, furfural 100–300 ppm, hydroxy methyl furfural 100–500 ppm, galactose 0 %, mannose 0 % and phenolics 2500–4000 ppm.

### Microorganism and inoculum cultivation

*Candida tropicalis* NCIM 3123 was purchased from National Collection of Industrial Microorganism (NCIM), Pune and maintained on MXYP agar slants (containing malt extract, 3 g/L; xylose, 20 g/L, yeast extract, 3 g/L, peptone, 5 g/L and agar, 20 g/Lat pH 7.0) at 4 °C. The microorganism was sub cultured every 2 weeks. A loopful of *C. tropicalis* strain from the 24-h-old slants maintained on MXYP agar slants were inoculated into 50 mL of inoculum medium containing (g/L) xylose, 20.0; yeast extract, 3.0; malt extract, 3.0; and peptone, 5.0 at pH 6.5 in 250 mL Erlenmeyer flask and was allowed to incubate for 24 h at 30 °C with agitation at 150 rpm. Harvested culture broth was centrifuged at 10,000 rpm for 10 min at 4 °C temperature. The supernatant was removed and the cell pellet was washed twice with sterile distilled water and further used for immobilization studies.

### Preparation of immobilized cells

#### Entrapment in sodium alginate

An adequate volume of cell suspension was added to sterilized 20 mL of 4 % (w/v) sodium alginate prepared in distilled water to get cell concentration of 6 g/L. The slurry was extruded through a syringe into 0.2 M calcium chloride solution with constant stirring. The beads were allowed to cure in 0.2 M calcium chloride solution for 24 h at 4 °C followed by washing with sterile distilled water 3–4 times, and they were preserved in sterile distilled water until use.

#### Entrapment in polyvinyl alcohol

Sterile aqueous solution containing 2 % w/v sodium alginate and 11 % w/v polyvinyl alcohol were mixed with the thick cell suspension so as to reach a cell concentration of 6 g/L (dry weight). The mixture was then passed through syringe needle (18 gauge) into calcium chloride (40 g/L) with use of peristaltic pump. The beads were maintained in the calcium chloride solution at 4 °C for 3 h. Afterwards, they were washed with sterile distilled water.

#### Entrapment in agarose gel

An adequate yeast cell suspension was added to the sterile 3 % w/v agarose solution maintained at 40 °C to reach concentration of 6 g/L, mixed well with stirring and poured into sterile petri plate and allowed to solidify. The resultant block was cut into equal size cubes and added to sterile distilled water and kept in refrigerator (1 h) for curing. After curing, sterile water was decanted and the cubes were then washed 3–4 times with sterile distilled water and stored in same for further use.

#### Whole cell immobilization in polyacrylamide

An adequate yeast cell suspension was mixed with 15 mL of 12 % w/v polyacrylamide solution, 15 mL of distilled water, 3 mL sterile water, 200 μL ammonium persulphate (0.4 gm/mL), and 50 μL TEMED to reach cell concentration of 6 g/L and allowed to polymerize on a sterile petri plate for 1 h. The polymer sheet was cut into cubes (4 mm), rinsed in sterile distilled water and stored at 4 °C for curing for 3 h. The cubes were then washed 3–4 times with sterile distilled water and stored in same till further use.

#### Whole cell immobilization in gelatin

An adequate volume of yeast cell suspension was added to 15 mL of 6 % w/v sterile gelatin maintained at 45 °C, and poured into a sterile petri dish. The gel was over layered with 10 μL of 5 % w/v glutaraldehyde for hardening at 30 °C. The resulting block was cut into small-size cubes (4 mm) and the cubes were washed thoroughly with sterile distilled water for complete removal of excess glutaraldehyde. The cubes were then washed 3–4 times with sterile distilled water and stored in same till further use.

#### Whole cell immobilization in *κ*-carrageenan

Saline solution containing 4 % w/v *κ*-carrageenan was heated to 60 °C for complete dissolution, and allowed to cool at 40 °C. Then, it was mixed with an adequate volume of yeast cell suspension to get cell concentration of 6 g/L at 32–35 °C without affecting cell viability. The mixture was pumped slowly to 2 % w/v KCl solution to induce gelation. The gel were then washed 3–4 times with sterile distilled water and stored in sterile water till further use.

### Screening of different matrices for immobilization of *Candida tropicalis* cells for xylitol production

The fermentation performance and bead integrity evaluation was performed by direct contact with non-detoxified stream of corn cob pentose hydrolysate. Fermentation was carried out in 500 mL Erlenmeyer flask containing 200 mL of fermentation media. This media consists of xylose concentration of 40–60 g/L for respective stream with 500 ppm urea as nutritional component. Initial pH of media was adjusted to 6.5 using calcium hydroxide. The fermentation was carried out on rotary shaker at 150 rpm and 30 °C. The quantity of immobilised beads was added as inoculum in different flask so that each flask contains equal number of cells like that of free cell fermentation as control.

### Optimization of immobilization condition in sodium alginate by statistical method

The yeast cells were immobilized by encapsulation in calcium alginate beads by use of freezing–thawing method (Levia et al. 2013). An adequate volume of cell suspension was added to sterile 20 mL of 20, 30 and 40 g/L of sodium alginate prepared in distilled water to get final cell concentration of 6 g/L. The slurry was extruded through a syringe into calcium chloride solution of 10, 20 and 30 g/L with constant stirring. The beads were allowed to cure in calcium chloride solution for 24 h at 4 °C followed by washing with sterile distilled water 3–4 times, and submitted to the freezing–thawing cycles at −20 °C. The levels for each variable used are shown in Table 1.

**Table 1:** Experimental matrix of full factorial design of experiments and xylitol yield (*Y*_p/s_), volumetric productivity (*Q*_P_) and encapsulation efficiency in the pellet (EE), after 96-h fermentation according to 2^3^ full factorial design

Test run	Coded variable			Response		
	SA (%)	CC (%)	FTN (no. of cycles)	*Y*_p/s_ (g/g)	*Q*_P_ (g/L/h)	EE (%)
1	−1 (2.0)	−1 (1.0)	−1 (2)	0.61 ± 0.01	0.26 ± 0.01	99.21 ± 0.42
2	+1 (4.0)	−1 (1.0)	−1 (2)	0.52 ± 0.02	0.18 ± 0.02	99.44 ± 0.20
3	−1 (2.0)	+1 (3.0)	−1 (2)	0.58 ± 0.01	0.24 ± 0.01	99.67 ± 0.23
4	+1 (4.0)	+1 (3.0)	−1 (2)	0.56 ± 0.02	0.20 ± 0.01	99.49 ± 0.26
5	−1 (2.0)	−1 (1.0)	+1 (4.0)	0.74 ± 0.01	0.40 ± 0.00	99.94 ± 0.04
6	+1 (4.0)	−1 (1.0)	+1 (4)	0.71 ± 0.01	0.39 ± 0.0	99.59 ± 0.12
7	−1 (2.0)	+1 (3.0)	+1 (4)	0.71 ± 0.01	0.40 ± 0.01	99.37 ± 0.05
8	+1 (4.0)	+1 (3.0)	+1 (4)	0.71 ± 0.01	0.40 ± 0.00	99.37 ± 0.40
9	0 (1.0)	0 (2.0)	0 (3)	0.50 ± 0.01	0.16 ± 0.01	99.66 ± 0.36
10	0 (1.0)	0 (2.0)	0 (3)	0.51 ± 0.01	0.17 ± 0.01	99.67 ± 0.23
11	0 (1.0)	0 (2.0)	0 (3)	0.51 ± 0.01	0.18 ± 0.01	99.76 ± 0.22

*SA* sodium alginate, *CC* calcium chloride, *FTN* freezing–thawing cycle no. Yield (*Y*_p/s_); productivity, *Q*_P_ (g/L/h); Immobilization efficiency, EE (%)

A 2^3^ level full factorial design with 8 axial point and 3 replicates at the center point with total of 11 experiments was employed (Box et al. 1978) This design was used to select the optimal immobilization conditions like concentration of sodium alginate, calcium chloride and number of freezing–thawing cycles, to enhance the fermentation performance, bead integrity and to reduce mass transfer limitation. As mentioned in Table 1, selected three independent factors were controlled at two levels (−, +), namely 20 and 40 g/L for the sodium alginate concentration (SA), 10 and 30 g/L for the calcium chloride concentration (CC), and (2 and 4) for the number of freezing–thawing cycles (FTN). These factors and levels were suggested by previous results obtained with *Candida* spp. cell and yeast cells immobilized onto other supports, polyvinyl alcohol (Cunha et al. 2007, 2009), polyurethane foam (Wang et al. 2009) calcium alginate (Carvalho et al. 2005; Milessi et al. 2013; Chen et al. 2012; Lotfipour et al. 2012) under different conditions.

### Fermentation media conditions for statistical design of experiments

The non-detoxified stream was heated at 80 °C for 10 min after pH adjustment to 6.5 with calcium hydroxide and supplemented with 500 ppm urea as a nitrogen source. Batch fermentation were carried out in duplicate in Erlenmeyer flask containing 100 mL fermentation media with corn cob hydrolysate in 250 mL flask and 15 g of wet beads containing cells. The flasks were maintained on rotary shaker at 150 rpm and 30 °C. The effects of the immobilization variables on xylose-to-xylitol conversion as well as on the encapsulation efficiency by the support were investigated through statistical concepts using the Statgraphics program (version 16.0). To this purpose, the yield of xylitol on consumed xylose (*Y*_p/s_), the xylitol volumetric productivity (*Q*_P_), and the encapsulation efficiency (EE) were selected as the responses. *Y*_p/s_ was calculated as the ratio of amount of xylitol produced at the end of fermentation to the amount of xylose consumed, *Q*_P_ as the ratio of maximum xylitol concentration to the fermentation time, The immobilization efficiency (*Y*_i_, %) was calculated as the ratio of concentration of immobilized cells (*X*_i_, g/L) to the concentration of total (suspended plus immobilized) cells (*X*_T_, g/L) and multiplying by 100.

The fermentation efficiency of xylose to xylitol was calculated as the ratio of the net amount of xylitol produced (g/L) to the initial xylose (g/L) of the medium, The conversion efficiency was calculated as the ratio of difference between initial xylose (g/L) and the residual xylose (g/L) to the initial xylose (g/L) in the medium.

### Performance evaluation studies in shake flask

The calcium alginate beads were prepared using the optimum concentration of sodium alginate 20 g/L, calcium chloride 10 g/L and 4 number of freezing–thawing cycles at −20 °C. The fermentation performance was evaluated in 500 mL shake flask containing 200 mL of non-detoxified hydrolysate media (55–60 g/L of xylose, 500 ppm urea and pH was adjusted to 6.5 with calcium hydroxide). Media was sterilized at 80 °C for 10 min in a vertical autoclave (Equitronics, Media instruments, Mumbai). Media was inoculated with 15 g of wet beads and operated at agitation of 150 rpm and temperature of 30 °C. Samples were periodically removed and analyzed for xylose and xylitol.

### Recyclability study of beads in non-detoxified pentose hydrolysate

The treated hydrolysate was heated at 80 °C for 10 min and supplemented with urea (0.5 g/L), before being used as a fermentation medium. pH was adjusted to 6.5 with calcium hydroxide. Duplicate repeated-batch fermentation runs were carried out in 250-mL Erlenmeyer flasks containing and 100 mL of fermentation medium and 10 g of immobilized biocatalysts. The flasks were maintained in a rotary shaker at 150 rpm and 30 °C for 96 h. After each run/cycle, the fermented medium was discarded, the solid carrier with immobilized cells were carefully drained and gently washed with water to eliminate all non-adhering yeast cells and the immobilized biocatalysts were charged with fresh medium. At the end of each cycle amount of xylitol produced was estimated and the process was carried out using the same immobilized cells for successive cycles.

### Analysis

Cell concentration in the resulting suspension was determined by optical density (OD) measurements at 640 nm, using a spectrophotometer, model U-2900 (Hitachi, Tokyo, Japan). The control was the fermentation medium without cells and corn cob hydrolysate particles to avoid any interference of solids on the OD measurements. The immobilized cell concentration was estimated by the same method after dissolution of beads in 0.1 M sodium citrate solution.

A previously constructed calibration curve was used to relate the OD measurements to dry cell concentration in samples of both this suspension and those used for inoculum. Sugar and xylitol concentrations were measured by HPLC, system of Agilent Technologies, 1200 series, model 1200 series (Agilent technologies, CA, USA), equipped with an Aminex HPX-87H (300 × 7.8 mm) column (Bio-Rad, Hercules, CA) and a refractive index G 1362 A detector, an G 1316 B column Oven and G 1311 A pump. Samples were previously filtered through 0.22 µ filter and injected in the column under the following conditions: injection volume of 20 µL, column temperature of 45 °C, 0.05 M H_2_SO_4_ as the mobile phase used at a flow rate of 0.6 mL/min.

## Results and discussion

In this study, yeast immobilization conditions in calcium alginate were established through the factorial design as mentioned earlier. The research work is distributed in three stages. The first stage involved screening of various matrices to produce xylitol by fermentation in non-detoxified corn corb hydrolysate and to select the matrix with best xylitol fermentation efficiency and stability of matrices in maintaining the shape in corn cob hydrolysate media. Second stage dealt with influence of sodium alginate concentration, calcium chloride concentration and number of freezing–thawing cycles on xylitol yield (*Y*_p/s_), volumetric productivity (*Q*_P_) and encapsulation efficiency (EE) by the support at shake flask level fermentation study and third stage studied the fermentation using immobilized beads prepared using statistically optimized conditions.

### Screening of various matrices used for immobilization for *Candida tropicalis* in non-detoxified corn cob hydrolysate

**Fig. 1:**
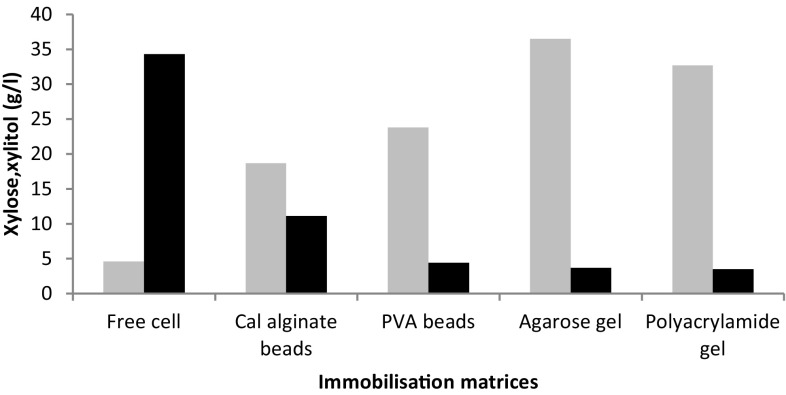
Comparative fermentation study of various matrices in relation to residual xylose and xylitol produced after 96 h in non-detoxified hydrolysate. *Black bar* xylose and *grey bar* xylitol

Figure 1 represents comparative study of stability and fermentation efficiency of immobilized beads or gel with *C. tropicalis* cells in non-detoxified corn cob hydrolysate for xylitol production. Polymers like sodium alginate, polyvinyl alcohol, agar, gelatin, polyacrylamide and *κ*-carrageenan were used as immobilization matrices to form beads or gels. Out of these polymers, beads were formed with sodium alginate, polyvinyl alcohol and gel formation done with agar, gelatin, polyacrylamide and *κ*-carrageenan. Shake flask fermentation study with these matrices in non-detoxified corn cob hydrolysate resulted into dissolution of gels like gelatin and *κ*-carrageenan just after addition. Polyvinyl alcohol bead, agarose and polyacrylamide gels were dissolved in non-detoxified media in first cycle. Only calcium alginate beads were not dissolved and maintained their spherical shape even after first cycle of reaction. Higher xylitol fermentation efficiency of 30 %, observed with calcium alginate as compared to all other polymers. Further optimization was required to match the efficiency of free cells used as control. Out of these four polymers, calcium alginate beads were selected for further study and optimization due to their integrity, stability, reusability and high fermentation efficiency among all supports.

### Beads preparation and fermentation performance evaluation

Bead preparation was carried out using full factorial design. The obtained responses indicated that the beads obtained in each run were effective to perform the bioconversion and to maintain the integrity in non-detoxified corn cob hemicellulosic hydrolysate. One of the important point observed was that encapsulation efficiency (EE) observed in each run was more than 99.0 %. The fermentation performance obtained for each run was as shown in Table 1. The xylitol yield, volumetric productivity, and encapsulation efficiency within the pellet were found to be varying in the ranges 0.40–0.74 g/g, 0.17–0.40 g/L^/^h, and 99.21–99.94 %, respectively.

### Statistical data analysis

**Fig. 2:**
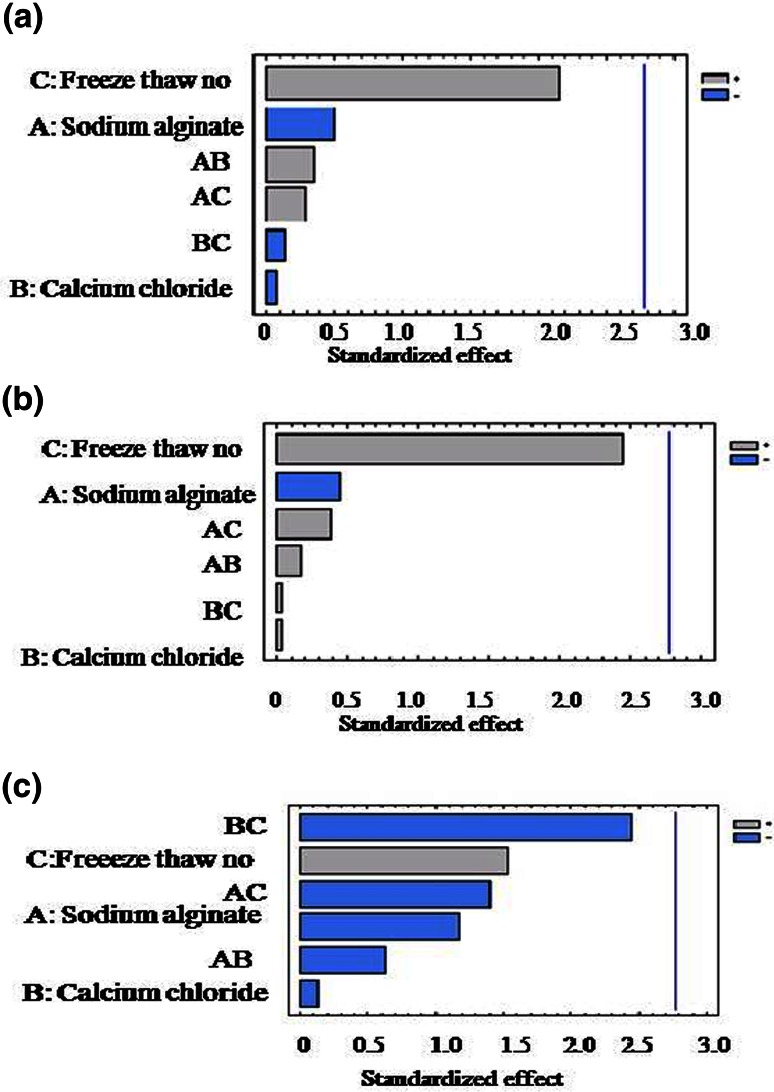
Pareto charts of independent variables and their interaction on each response variable. **a** Yield, **b** productivity, **c** immobilization efficiency

Figure 2 shows the Pareto charts for each response variable. From analysis of Pareto charts and Anova table it can be concluded that xylitol yield, productivity and immobilization efficiency were not influenced by sodium alginate, calcium chloride concentration and freezing–thawing cycle number (*p* value > 0.05). The *p* value revealed that xylitol yield and xylitol productivity was only moderately influenced by the freezing–thawing cycle number (80 % < significance < 95 %). Although a very negligible effect of calcium chloride was observed on yield, productivity and immobilization efficiency, moderately significant interactive effect of calcium chloride and freeze thaw cycle no was observed on immobilization efficiency (80 % < significance < 95 %). Insignificant interactive effects were observed for yield and productivity. The optimum levels for the variables obtained by use of statgraphics software were sodium alginate (2 % w/v), calcium chloride (1.0 % w/v) and 4 number of freezing and thawing cycle.

### Fermentations with immobilized cell pellet in shake flask

**Fig. 3:**
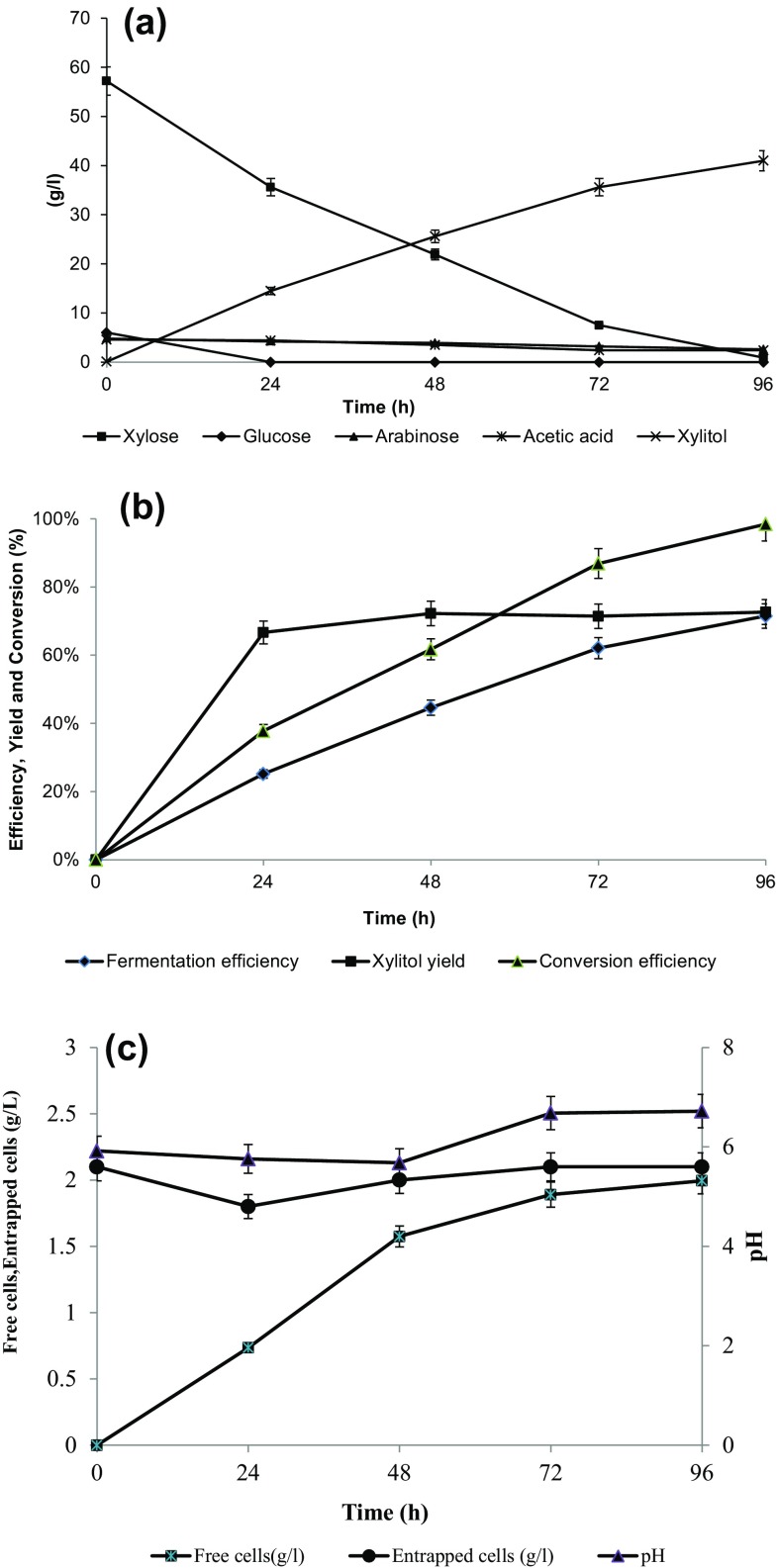
Fermentation profile of xylose to xylitol using optimized immobilized beads. **a** Consumption of xylose, arabinose, glucose and acetic acid as well as xylitol production. **b** Profile of fermentation performance with immobilized bead in shake flask (initial xylose, 57.2 g/L; pH 5.92; Temperature 30 °C). **c** Concentration profile of free cells, cells immobilized in the calcium alginate and pH during fermentation of corn cob hemicellulosic hydrolysate. *Error bars* represent variation between the duplicate trials

Figure 3a shows the consumption pattern for different sugars like glucose, xylose, arabinose, acetic acid and xylitol production during fermentations performed in shake flask on a medium based on corn cob hemicellulosic hydrolysate and with pellets prepared using sodium alginate concentration of 20 g/L, calcium chloride concentration of 10 g/L and four freezing–thawing cycles at freezing temperature of −20 °C.

As shown in the Fig. 3a glucose was the preferred substrate as compared to xylose. Glucose was consumed within first 12–24 h only. The presence of glucose is responsible for biomass formation (Yahashi et al. 1996), ethanol formation and known to regulate xylitol formation in pentose fermenting yeasts as it represses the synthesis of xylose reductase (Kastener et al. 2001) the main enzyme responsible for xylose-to-xylitol reduction.

As can be observed in Fig. 3a, b, *C. tropicalis* cells immobilized in sodium alginate beads were able to convert xylose to xylitol during the fermentation in shake flask, consuming around 98 % xylose and accumulating 41 g/L xylitol after 96 h. The values of xylitol yield (0.73 g/g) and volumetric productivity (0.43 g/L^/^h) obtained in the present work (Table 2) is better than most of the similar studies based on corn cob hydrolysate (Wang et al. 2009; Cheng et al. 2009; Bibbins et al. 2013). El-Batal and Khalaf (2004), reported higher xylitol titer (48 g/L) than the present work. However, the yield was low and the hydrolysate was detoxified prior to fermentation. Production of xylitol with corn cob hydrolysate is usually less as compared to rice straw hydrolysate (Table 3).

**Table 2:** Results of xylose-to-xylitol bioconversion by *C. tropicalis* cells immobilized in calcium alginate beads after 96 h of cultivation in medium based on corn cob hemicellulosic hydrolysate

Percentage of xylose consumption (%)	98
Final xylitol concentration (g/L)	41
Yield of xylitol on consumed xylose (g/g)	0.73
Xylitol productivity (g/L/h)	0.43

**Table 3:** Comparison with other *Candida* sp. and yeast fermentation performance

Microorganism	Feedstock/Method of detoxification	Xylitol production			
		Concentration (g/L)	Yield (g/g)	Productivity (g/L/h)	References
*C. tropicalis* in Poly urethane foam	Corn cob hydrolysate, detoxification with lime	–	0.66	1.90	Wang et al. (2009)
Hydrogel copolymer immobilized *C. tropicalis*	Corn cob hydrolysate with detoxification by resin and charcoal treatment	48.5	0.58	–	El-Batal and Khalaf (2004)
*Debaromyces hansenii* in Calcium alginate beads	Crude corn cob hydrolysate without detoxification (shake flask)	12.9	0.53	0.23	Bibbins et al. (2013)
*C. guillermondii* in calcium alginate	Corn cob hydrolysate with calcium oxide treatment and charcoal treatment	–	0.58	0.39	Cheng et al. (2009)
*Candida tropicalis* CCTCC M2012462	Corn cob hemicellulosic hydrolysate without detoxification	38.8	0.70	0.46	Ping et al. (2013)
*Candida subtropicalis* in polyacrylic hydrogel	Rice straw hydrolysate, detoxified with Calcium oxide and charcoal treatment	–	0.73	–	Liaw et al. (2008)
*C. tropicalis* in Ca-alginate	Rice straw hydrolysate with lime treatment and charcoal treatment	74	0.73	0.533	Deng et al. (2006)
*Candida tropicalis* JH030	Rice straw hydrolysate without detoxification	31.1	0.71	0.44	Huang et al. (2011)
*C guillermondii* FTI 20037	Rice straw hydrolysate without detoxification	50	0.67	0.41	Silva and Roberto (2001)
*Candida tropicalis* in Ca-alginate beads	SCB hydrolysate with detoxification	21	0.54	0.44	Carvalho et al. (2003)
*C. guillermondii* FTI 20037 in calcium alginate	Sugar cane bagasse hemicellulosic hydrolysate detoxification with ion exchange resin	–	0.62	0.24	Carvalho et al. (2004)
*C guillermondii* in calcium alginate	Sugarcane bagasse hydrolysate with detoxification	28.9	0.58	0.40	Sarrouh et al. (2007)
*Debaromyces hansenii free cells*	SCB hydrolysate with charcoal and ion exchange resin	–	0.69	0.28	Gyan et al. (2011)
*C. tropicalis* in Calcium alginate–chitosan	Poplar wood chips concentrated and neutralised	–	0.40	–	Jing et al. (2009)
*Candida tropicalis* in calcium alginate beads	Corn cob hydrolysate without detoxification (optimized condition)	41	0.73	0.43	This study

Arabinose consumption was slow as compared to xylose, only 40 % of initial arabinose was consumed in 96 h. Same phenomenon was reported by other authors also (Yahashi et al. 1996) Acetic acid level at the beginning of this fermentation (4.6 g/L) was decreased to 2.4 g/L at the 96 h of fermentation. Overall acetic acid was not consumed as initial concentration was more than 2 g/L. Morita and Silva (2000), reported that at acetic acid concentration (>1.0 g/L), part of acid continues to be directed towards the Krebs cycle and the remainder may be utilized by another energy consuming metabolic pathway (Morita and Silva 2000). Cheng et al. (2009) studied the effect of glucose and acetic acid in the corn cob hydrolysate on xylitol production with *C. tropicalis*, Biomass growth favored by glucose but acetic acid at high concentration (>2 g/L) was inhibitory. Hence this strain was found to be more efficient to consume acetic acid (Cheng et al. 2009).

Cell growth was considered as the increase of the free cells concentration (*X*_f_) in the fermentation medium and of immobilized cells in the gel beads (*X*_i_). Figure 3c shows that the final concentration of free cell and the immobilized cell concentration at every periodic hours. Immobilized cell concentration decreased up to 24 h and then increased up to 48 h and remained almost constant until the end of the fermentation. Free cell concentration, which shows around 50–60 % of the total biomass at the end of the fermentation. This result suggests that the pores of the gel matrix were saturated by immobilized cells after this short time. A portion of these cells was released from the beads and proliferated in the medium; reaching a maximum concentration of 95 × 10^6^ cells/mL. Similar results were obtained by other researchers also (Bibbins et al. 2013).

Hence, the studied fermentation occurred due to both free cells and immobilized cells, since a fraction of cells was freely suspended in the medium and another one was entrapped in the beads. The coexistence of mixed cultures consisting and free and immobilized cells is also described by several authors working on different immobilization systems **(**Bibbins et al. 2013; El-Batal and Khalaf 2004 **).** This phenomenon might be due to two factors mainly cell leakage from the immobilized beads due to the shearing forces during agitation and the preferential growth of free cells generated from the surface of bead due to favorable substrate and medium conditions. Liouni et al. (2008), attributed the ability of cells located on the periphery of single cells to multiply and released into suspension as free cells. After 72 h of fermentation, free cells concentration maintained almost constant, probably due to oxygen limitation conditions created either by high biomass level or oxygen depletion in the broth. After this period, xylitol accumulated in the medium and reached its maximum concentration (41 g/L) at the end of the run.

Till now, several research studies have been done with hemicellulosic acid hydrolysate from various lignocellulosic materials for xylitol production. A summary of these results are listed in Table 3. A number of previous studies focus only on detoxification of hydrolysate either by over liming (Wang et al. 2009) or combination with other detoxification method such as activated charcoal (Cheng et al. 2009, Liaw et al. 2008) or ion exchange (Carvalho et al. 2004, Gyan et al. 2011) to improve fermentation performance. Detoxification helps to remove toxic inhibitors like phenolics, furfural and 5-hydroxy methyl furfural (HMF). However, detoxification resulted into 5–10 % loss of sugar which adds to process cost. Only few studies have focused on fermenting hydrolysate without detoxification (Huang et al. 2011; Misra et al. 2013; Ping et al. 2013). In the relevant literature collected, the type of the hydrolysate discussed in the studies of converting xylose into xylitol by microorganisms include corn cob, corn fiber, sugarcane bagasse, hardwood, eucalyptus, rice straw, etc. Among which there is a large difference in relevant xylitol yield. The maximum yield from these studies varies widely from 0.40 to 0.73 g/g and productivity from 0.24 to 1.92 g/L/h.

Bibbins et al. (2013), obtained, fermentation of *Debaromyces hansenii* immobilized in calcium alginate beads in non-detoxified corn cob hydrolysate, resulted into 12.9 g/L of xylitol, 0.53 g/g yield and 0.23 g/L/h productivity in shake flask with use of synthetic nutrients and Misra et al. (2013), studied scale up in the 10 L reactor with working volume of 5 L of corn cob hemicellulosic hydrolysate without detoxification and adapted *C. tropicalis* cells to yield product titer of 11.89 g/L of xylitol, yield of 0.58 g/g, volume productivity 0.28 g/L/h and efficiency of 63.73 %. Kwon et al. (2006) used cell recycle fermentation using hollow fiber membrane to get xylitol productivity of 4.14 g/L/h and 0.82 g/g yield with synthetic pure xylose and Kim et al. (2004) produced xylitol from xylose by cell recycle fermentation of *C. tropicalis* using chemically defined medium to get 5.4 g/L/h productivity and 0.81 g/g yield. Whereas in this study yield, productivity and xylitol titer of 0.73 (theoretically 0.80) g/g, 0.43 g/L/h, 42 g/L were obtained, respectively from corn cob hydrolysate (hydrolysate contained 55–60 g/L xylose, 4.6 g/L acetic acid, 0.30 g/L of furfural and 0.20 g/L of 5-HMF) without any detoxification of hydrolysate or purification of xylose.

### Reuse of immobilized cells

**Fig. 4:**
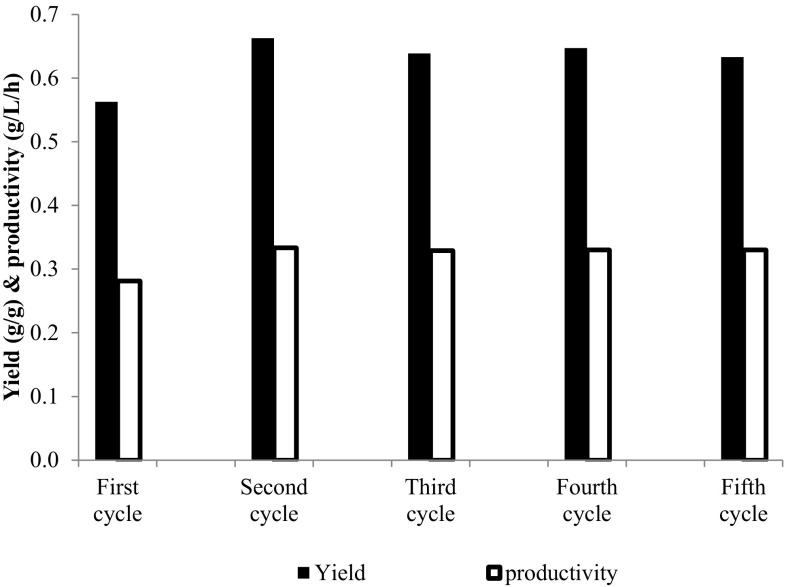
Repeated batch xylose-to-xylitol bioconversion with cell recycling (initial xylose: 52 g/L; temp: 30 °C, agitation: 150 rpm)

*Candida tropicalis* cells were immobilized in Ca-alginate matrix as described in “Materials and methods”. Immobilized cells were used as inoculums for recycling batches. Such reuse was performed five times without impairment in the bioconversion rates and yields. The immobilized yeast produced xylitol with an average productivity of 0.32 g/L/h and theoretical conversion efficiency of 92 % for five successive batches (Fig. 4). The possibility of producing xylitol with Ca-alginate entrapped cells in synthetic xylose solutions (Misra et al. 2013; Carvalho et al. 2003) and in lignocellulosic hydrolysate (Misra et al. 2013) has been previously demonstrated.

*Candia tropicalis* NCIM 3123 used in this study showed good potential for xylitol production in producing good xylitol yield and concentration in non-detoxified hydrolysate. More importantly, there was no breakage or disruption of calcium alginate beads in fermentation, so such cells of *C. tropicalis* immobilized in calcium alginate showed high stability and high performance for xylitol production from corn cob hemicellulosic hydrolysate. The preliminary study presented here demonstrate that the potential of high inhibitor tolerance of *C. tropicalis* NCIM 3123 when utilized with non-detoxified hydrolysate. In the future optimization of fermentation condition and media optimization for the yeast needs to be investigated.

## Conclusions

Industrially feasible process for production of xylitol from non-detoxified hydrolysate with use of entrapped *C. tropicalis* is demonstrated. *C. tropicalis* cells were successfully immobilized in calcium alginate using the freezing–thawing method using a 2^3^ full factorial design. More than 99.50 % immobilization efficiency obtained with beads maintaining size, shape in hydrolysate without any visible wearing. The results obtained in terms of the yield of xylitol on consumed xylose, xylitol volumetric productivity, and immobilization efficiency suggested a sodium alginate concentration of 20 g/L, calcium chloride concentration of 10 g/L and 4 numbers of freezing–thawing cycles at freezing temperature of −20 °C as the most optimized conditions for pellet preparation. Reused immobilized biomass showed sustained xylitol production even after 5 cycles. These results demonstrate the feasibility of the proposed immobilization system to be used in future industrial xylose-to-xylitol production from low cost hemicellulosic hydrolysate.

## Electronic supplementary material

Below is the link to the electronic supplementary material.

Supplementary material 1 (DOCX 26 kb)
