# Supplementary material for: Enhanced xylitol production using immobilized Candida tropicalis with non-detoxified corn cob hemicellulosic hydrolysate
Source: 3 Biotech. 2016 Feb 16;6(1):75. doi: 10.1007/s13205-016-0388-8 (PMC4755960; doi:10.1007/s13205-016-0388-8)

**Table 1 Effect of each process variable on response variable**

| Factor | Estimated effects of process variable on response variables | | |
| --- | --- | --- | --- |
|  | Yp/s | Productivity | Immo Efficiency |
| SA | -0.035 | -0.0325 | -0.13 |
| CC | -0.005 | 0.0025 | -0.015 |
| FTN | 0.15 | 0.1775 | 0.17 |
| SA X CC ^a^ | 0.025 | 0.0125 | -0.07 |
| SA X FTN ^a^ | 0.02 | 0.0275 | -0.155 |
| CCX FTN ^a^ | -0.01 | 0.0025 | -0.27 |

a : Interaction between factors

**Table 2 Analysis of variance and significance of the process variable for response variables**

| **Xylitol yield (Yp/s)** | | | | | |
| --- | --- | --- | --- | --- | --- |
| **source** | **Sum of squares** | **df** | **Mean square** | **F-ratio** | **p-value** |
| **SA** | 0.00245 | 1 | 0.00245 | 0.25 | 0.6404 |
| CC | 0.00005 | 1 | 0.00005 | 0.01 | 0.9460 |
| FTN | 0.045 | 1 | 0.045 | 4.67 | 0.0967 |
| SA X CC ^a^ | 0.00125 | 1 | 0.00125 | 0.13 | 0.7368 |
| SA X FTN ^a^ | 0.0008 | 1 | 0.0008 | 0.08 | 0.7875 |
| CCX FTN ^a^ | 0.0002 | 1 | 0.0002 | 0.02 | 0.8924 |
| **Total error** | 0.0385045 | 4 | 0.00962614 |  |  |
| **Total (Corr)** | 0.0882545 | 10 |  |  |  |
| Xylitol productivity (Qp) | | | | | |
| **SA** | 0.0021125 | 1 | 0.0021125 | 0.20 | 0.6774 |
| CC | 0.0000125 | 1 | 0.0000125 | 0.00 | 0.9742 |
| FTN | 0.0630125 | 1 | 0.0630125 | 5.98 | 0.0707 |
| SA X CC ^a^ | 0.0003125 | 1 | 0.0003125 | 0.03 | 0.8716 |
| SA X FTN ^a^ | 0.0015125 | 1 | 0.0015125 | 0.14 | 0.7239 |
| CCX FTN ^a^ | 0.0000125 | 1 | 0.0000125 | 0.00 | 0.9742 |
| **Total error** | 0.0421159 | 4 | 0.010529 |  |  |
| **Total (Corr)** | 0.109091 | 10 |  |  |  |
| Immobilisation efficiency (EE) | | | | | |
| **SA** | 0.0338 | 1 | 0.0338 | 1.38 | 0.3049 |
| CC | 0.00045 | 1 | 0.00045 | 0.02 | 0.8986 |
| FTN | 0.0578 | 1 | 0.0578 | 2.36 | 0.1990 |
| SA X CC ^a^ | 0.0098 | 1 | 0.0098 | 0.40 | 0.5610 |
| SA X FTN ^a^ | 0.04805 | 1 | 0.04805 | 1.97 | 0.2336 |
| CCX FTN ^a^ | 0.1458 | 1 | 0.1458 | 5.96 | 0.0710 |
| **Total error** | 0.0977909 | 4 | 0.0244477 |  |  |
| **Total (Corr)** | 0.393491 | 10 |  |  |  |

a two factor interactions

b Significance≥95%

c Significance<80%

d 80≤Significance<90%

e 90≤Significance<95%

Fig 1: Tendency of each response variable with the range of process variables (positive and negative levels).


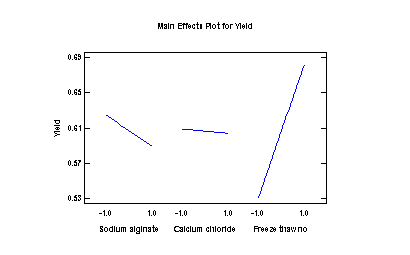

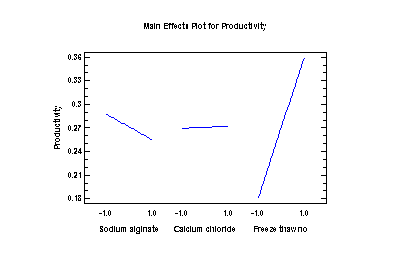


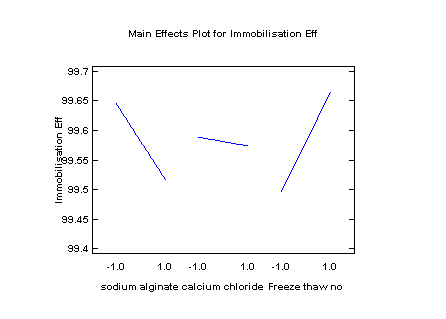

Supplement: Supplementary file 1 — Supplementary material 1 (DOCX 26 kb) [file 13205_2016_388_MOESM1_ESM.docx]
